# Supplementary material for: Perceptions and Practices of General Practitioners towards Oral Cancer and Emerging Risk Factors among Indian Immigrants in Australia: A Qualitative Study
Source: Int J Environ Res Public Health. 2021 Oct 22;18(21):11111. doi: 10.3390/ijerph182111111 (PMC8582889; doi:10.3390/ijerph182111111)
Supplement: Supplementary file 1 [file ijerph-18-11111-s001.zip › ijerph-1397716-supplementary/Supplementary_file_2_revised_Interview guide.pdf]

Supplementary file 2: Interview guide (and guiding question/prompts)

Interview protocol:

- Talk to the participants (GPs).
- Confirm that participants have read the participant information sheet and is consenting for the interviews to be recorded.
- Ask if they have any other questions.
- Provide a brief summary of the project and state the main purpose of the interview.

Focus areas (to be explored by interview)

- Background information of participant/GP.
- Knowledge and beliefs of GPs regarding oral cancer risk among Indian immigrants in Australia.
- Current clinical practices regarding oral cancer risk assessments.
- Possible barriers according to GPs in oral cancer awareness specially among Indian immigrants.
- Other comments/questions.

Guiding questions:

PROFESSIONAL BACKGROUND

1. How long you have been practicing in Australia?

2. Could you please tell me a little bit about professional education/ medical degrees you undertook?

3. Did you get your undergraduate degree within OR outside Australia?

4. Considering the location of this medical practice, I presume you deal with various population groups usually. Do you encounter with a lot of Indians here?

{ask GP to explain or give examples in case of brief answers}

KNOWLEDGE REGARDING ORAL CANCER

5. Could you please tell me a little about oral cancer, its signs/symptoms and the risk factors associated with it?

6. What are your thoughts in relation to risk products like smokeless tobacco, betel quid- can they cause oral cancer?

7.Do you have any idea if such products are available in Australia?

8.Are you aware of any population groups which are at high risk for oral cancer? (in Australia??)

9.Have you received any educational training/workshops/seminars regarding oral cancer screening/identification specially in relation to high-risk populations (like Indians??) {if yes, explain, if no???)

ATTITUDES/BELIEFS REGARDING ORAL CANCER

10. What are your thoughts about the current oral cancer situation in world and Australia? OR What do you think are factors behind that? (elaborate if time permits else next ques.)

11.Do you think GPs play an important role in identification of oral cancer?

12. If yes- what type of role?

If no- who do you think has more important role, then? Example-dentist??

13. Do you think that people/patients would be more comfortable while talking about oral cancer with a GP?

14. If you were to provide referral assessment, awareness/preventative education to patients, particularly Indians in Australia, do you think they would be receptive to that? Example?

PRACTICES REGARDING ORAL CANCER

15. Do you generally discuss about oral cancer with your patients OR has it come up during check-ups if initiated by patient?

16. If yes- elaborate or give example

If no- What do you generally do if you encounter a patient with history or complain of non-healing ulcer in palate or tongue? Referral??

17.Are you currently using any standard/non-standard risk assessment strategy in relation to oral cancer? If yes-what, how?

18.Do you ask about the risk habits during your usual check-up of patient specially Indians?

19. Do you have any resources available to motivate patients to talk/ask about oral cancer OR to educate them?

BARRIERS REGARDING ORAL CANCER ASSESSMENT

20. What do think about barriers in oral cancer assessment in GP practice, particularly in relation to Indian population?

21.Do you think some educational programs or special training webinars/seminars for GPs may help? If yes, what kind you would be interested in?

22. What are your suggestions regarding raising awareness about oral cancer among populations at high risk?

23. Do you think some resources like brochures, DVDs in waiting area or leaflets in your practice may help/prompt patients somehow? (depending on their reply in ‘practices’?)

24.Would you be interested in any kind of standard risk assessment tool for oral cancer specifically for Indian population? If yes, What kind? --------------Thanks… (stop recording)
